# Treatment of Chloroform Collapse

**Published:** 1894-03-31

**Authors:** Benjamin Ward Richardson


### Treatment of Chloroform Collapse.

By Sir Benjamin Ward Richardson, M.D., F.R.S.

Since the death by chloroform of Hannah Greener, at Winlaton, near Newcastle, on January 28th, 1848, recorded as the first death from chloroform administration, the utmost interest has attached itself to all methods of treatment intended to meet the fatal effects of chloroform, or, in other words, intended to produce resuscitation from chloroform collapse. I was one of the first to study this question, and in 1850, after a patient inquiry, had prepared an experimental essay on the subject, which led, very quickly, to a series of articles in the old *Medical Gazette*, "On the different modes in which death occurs." From then up to this time the subject has always been before me both in reading and in original research. As deaths from chloroform have of late been very frequent, beyond, in fact, anything in my recollection, I propose in this paper to touch on the means most likely to be of service in such catastrophes, which, as I computed in 1864, occur once in about 2,500 administrations, a calculation which experience has very severely confirmed.

When death from chloroform began to engage the attention of practitioners at first the means adopted for treatment were varied. Each man seemed to take his own bent. One employed artificial respiration; another galvanism; another trusted to pulling the tongue forward; a fourth to flipping the body with a towel; a fifth to applying ammonia to the nostrils; a sixth to dashing cold water in the face, and a seventh to brisk friction of the limbs and body. From then until now these methods have been principally relied upon by different practitioners according to their particular ideas and conveniences at the time of the accident. In brief, there has been no one particular method adopted uniform in its character and relied upon as to results. One method, perhaps, more than another, has, however, been on the whole valued, namely, artificial respiration. Snow, who never seems to have been present at a death from chloroform, was of opinion that artificial respiration was the one and sound mode of treatment, and there can be no doubt that his teaching influenced the profession from the first. But, unfortunately, the mode of carrying out artificial respiration varies so considerably, that each adoption of it becomes, as it were, an experiment which may in some cases be successful, but may in others have just the opposite effect. It has been towards a settlement of the difficulties surrounding all methods that my own researches have been directed throughout; and although I regret I have discovered little that can be said to be affirmative in action, I have certainly learned what is detrimental on one side and what is hopeful on the other.

My mode of research has consisted always in narcotising a warm-blooded animal quickly or slowly up to death, and after waiting until all signs of life have ceased, employing such means for resuscitation as had been in every case prepared for, with every appliance at hand for working out the intention. There has, therefore, never been any hurry in procedure, and I think I may safely say never any blundering or undecided change of method from anxiety, or interposition of new suggestion. I may also add that in every case actual death had so far occured, that if the animal had been left alone there was no probability of its spontaneous rally and recovery.

The upshot of all has been to show that artificial respiration conducted under certain favourable circumstances is the surest and best remedial measure. Every attempt made with galvanism and other forms of electrical current, while they sometimes led to specious appearances, as if vitality were returning, only seemed to clench, the fatal symptoms. Galvanization was carried out, as my published researches have shown, in the most varied manner for purposes of resuscitation. In some instances the muscles of respiration were made to act from measured shocks through a metronome; so that natural breathing seemed to be perfectly established under the current, but directly the current was stopped, all stopped; that is to say there was no return of natural breathing. Moreover, it was soon found that the muscular irritability of the diaphragm and other respiratory muscles speedily wore out so that an animal with galvanized muscles retained vitality of muscle a much shorter time than one that was left entirely untouched. This was a very striking fact. I made a pair of double-acting bellows for exhausting and injecting air into the lungs, and to this bellows I adjusted a galvanic apparatus which would allow me to set up a bellows respiration in combination with, and synchronous with the respiration produced by the current through the respiratory muscles. No apparatus could work better, but I found that when two animals dead from chloroform were treated, the one with the bellows simply without galvanism, the other with the galvanism superadded, recovery under the simple process would often occur, while recovery with the addition of the galvanism never once followed. The muscles were exhausted by the current, before they were re-supplied with blood and thereby recharged for contraction. In like manner I put to the test the influence of galvanism on the heart. Snow had suggested that galvanism might be of service in stimulating the heart into action if needles were inserted into the actual structure of the heart through the chest wall. I tested this suggestion in every conceivable form I could devise, but never attained to any result than that of apparently stopping the action of the heart prematurely. I mean that cardiac action which is not felt through the thorax, but which is visible as a minor action if the chest wall be removed, and the heart itself be observed with its auricles and ventricles contracting. I moved from this line of experiment to the observation of the effects of electric currents, intermittent, continuous, and frictional, on the heart itself while it was still contracting after death, and I found that although each current would, for the moment, excite contraction and increase it, it, without fail, shortened the time of contraction, so that the heart of the dead animal died sooner when its motion was exalted by any of these currents than when it was left to cease of itself. I found also that in the hearts of the cold-bloods, as turtles, which after death will continue to contract for several hours, the contractile power could always be brought to naught more speedily by electrical currents than by leaving the organ to die out naturally. Moreover, I saw that although a quickened motion of the heart, which lasted for a considerable time, could be induced by current stimulation, there was never sufficient power exerted to drive a column of blood from the right side of the heart over to the left, or from the left over the body by the aorta. Again, I could never establish true rythmical contraction of equal values between auricles and ventricles, so that in the end I could come to no other conclusion than that electric stimulation of the heart after death from chloroform is more than a mere delusion, it is a method by which more harm than good is likely to accrue.

